# Meta-analysis of the effect of curcumin supplementation on skeletal muscle damage status

**DOI:** 10.1371/journal.pone.0299135

**Published:** 2024-07-15

**Authors:** Xiaoyang Liu, Lihan Lin, Guopeng Hu

**Affiliations:** College of Physical Education, Huaqiao University, Quanzhou, Fujian, China; University of New Hampshire, UNITED STATES OF AMERICA

## Abstract

**Objectives:**

Meta-analysis was conducted to examine the effect of supplemental curcumin intake on skeletal muscle injury status and to propose an optimal intervention program.

**Methods:**

In accordance with the procedures specified in the PRISMA statement for systematic reviews and meta-analyses of randomized controlled trials, the Review Manager 5.3 was used to analyze the results of creatine kinase (CK), muscle soreness, interleukin-6 (IL-6), and range of motion (ROM) as outcome indicators in the 349 subjects included in the 14 articles.

**Results:**

The effect size of curcumin supplementation on muscle soreness, mean difference (MD) = -0.61; the relationship between curcumin supplementation and muscle soreness for time of measurement (I^2^ = 83.6%)、the relationship between curcumin supplementation and muscle soreness for period of intervention (I^2^ = 26.2%)、the relationship between whether one had been trained (I^2^ = 0%) and supplementation dose (I^2^ = 0%) were not heterogeneous for the relationship between curcumin supplementation and muscle soreness; The effect size on CK, MD = -137.32; the relationship between curcumin supplementation and CK (I^2^ = 79.7%)、intervention period (I^2^ = 91.9%)、whether or not trained (I^2^ = 90.7%)、and no heterogeneity in the relationship between curcumin supplementation and CK for the time of measurement (I^2^ = 0%); The effect size MD = 4.10 for the effect on ROM; The effect size for IL-6 was MD = -0.33.

**Conclusions:**

This meta-analysis highlights that curcumin supplementation significantly mitigates skeletal muscle damage, with notable improvements in CK levels, muscle soreness, IL-6 levels, and ROM. The results highlight the importance of curcumin dosage and timing, revealing that prolonged supplementation yields the best results, especially for untrained individuals or those less exposed to muscle-damaging exercise. For muscle soreness and ROM enhancement, a pre-emptive, low-dose regimen is beneficial, while immediate post-exercise supplementation is most effective at reducing CK and IL-6 levels.

## Introduction

Recovering from Exercise-Induced Muscle Damage (EIMD) is a significant challenge faced by athletes across various sports. Such injuries can hinder an athlete’s training progress and adversely impact overall performance. During exercise, mechanical damage to muscle fibers leads to degradation of muscle fibers and damage to Z-lines and T-tubules [1]. This damage impairs the regulation of calcium ion (Ca^2+^) release, contributing to further injury and eventual apoptosis of muscle fibers [2]. Consequently, this cell death sets off an inflammatory cascade that generating reactive oxygen species (ROS), which damages proteins and interferes with excitation-contraction coupling, ultimately resulting muscle performance [3]. The effects of EIMD includes diminished muscle strength, pronounced muscle soreness, elevated levels of oxidative stress and inflammation, and markers indicative of muscle damage [4].

Sports supplements have the potential to boost athletic performance and aid muscle recovery, but their use must be carefully integrated with a consistent exercise regimen. These supplements can lower energy expenditure, restore internal balance, promote nutrient regeneration, and ultimately enhance physical function [1,5]. Research has focused on curcumin’s potential to alleviate chronic inflammation and EIMD.

Curcumin is renowned for its myriad health benefits, including antioxidant, anti-inflammatory, and analgesic effects [6], which make it a highly popular supplement. Extracted from turmeric and approved for use as a food additive in China, curcumin activates SIRT1, potentially reducing inflammatory responses and protecting cardiovascular health. It also inhibits COX-2 and NF-kB signaling pathways while enhancing adaptability to training [7], offering similar benefits to NSAIDs without their side effects [8]. Its effectiveness in alleviating post-exercise inflammation has contributed to its growing popularity [9], though its exact mechanism of action remains unclear. A meta-analysis of studies in this field is necessary to derive more precise and reliable conclusions, ultimately providing a scientific basis for protecting athletes’ health.

When muscle tissue is damaged, creatine kinase (CK) is released into the bloodstream. Elevated CK levels are commonly recognized as a crucial biochemical marker of muscle damage and are typically proportional to the severity of the injury [10]. Thus, measuring CK levels provides a direct indication of muscle tissue damage.

Muscle soreness is a direct and subjective symptom experienced after muscle injury. The use of curcumin supplements, known for their anti-inflammatory properties, can reduce inflammation and relieve pain. Assessing pain levels, therefore, provides a direct reflection of curcumin’s potential benefits in enhancing muscle comfort.

Curcumin is known to potentially modulate the inflammatory response following muscle injury through its effects on the inflammatory cytokine interleukin-6 (IL-6). This cytokine plays a vital role in both muscle injury and repair processes [6]. Analyzing IL-6 expression provides insights into curcumin’s possible anti-inflammatory effects.

Range of motion (ROM) represents the maximum extent to which a joint can move freely [11]. Muscle damage often limits this mobility. Supplementation with curcumin could potentially improve muscle function and joint flexibility. Measuring ROM is an objective method for evaluating the effectiveness of muscle recovery interventions.

CK, IL-6 and ROM are recognized as standard indicators in clinical and sports medicine research. Their widespread use facilitates comparisons across studies and meta-analyses, thus increasing the reliability and validity of findings.

Collectively, these metrics capture a range of conditions from biochemical changes (CK, IL-6) to dysfunction (ROM) and subjective symptoms (muscle soreness), covering a broad spectrum of effects of muscle injury. This integrated approach provides a comprehensive view of muscle health and recovery. In addition, these markers are relatively easy to measure: CK and IL-6 by blood samples, and ROM and muscle soreness by non-invasive physical examination and self-report scales, respectively. This simplicity makes them ideal for studies with multiple time points or large sample sizes.

Incorporating these markers into studies of curcumin supplementation’s effects on skeletal muscle damage allows robust analyses of all aspects of the compound’s impact on muscle function and integrity. This not only helps to understand the underlying biochemical processes, but also helps to assess functional outcomes, which is essential for translating research findings into practical recommendations for exercise and health.

Based on the aforementioned factors, this study formulated the research hypothesis that curcumin supplementation is effective in alleviating muscle soreness, reducing in vivo CK levels, enhancing ROM, and IL-6 concentrations. To examine the impact of curcumin supplementation on muscle soreness relief and CK reduction in vivo, this study introduced four moderating variables: Time of Measurement: The included literature was categorized into six groups for analysis: pre-exercise, immediate post-exercise, 24 hours post-exercise, 48 hours post-exercise, 72 hours post-exercise, and 96 hours post-exercise. Training Status: The gathered literature was classified into two groups: trained and untrained, to investigate any potential variations in the effects of curcumin. Intervention Duration: The collected literature was subdivided into three groups based on the intervention period: single dose, less than one week, and one week or more, to assess the influence of different intervention durations. Supplement Dosage: The compiled literature was segregated into three groups concerning supplement dosage: <0.5g, 0.5–1.5g, and >1.5g, to explore the dose-dependent effects of curcumin supplementation.

## Study methodology

### Inclusion and exclusion criteria

#### Inclusion criteria

The articles must be based on randomized controlled trials (RCTs), with adult subjects, and using curcumin or a curcumin-based supplement as the focus. Primary outcome indicators should include CK, muscle soreness, IL-6, and ROM.

#### Exclusion criteria

Unclear study types, invalid outcome indicators, duplicate literature, conferences, and studies not in Chinese or English were excluded.

### Literature search strategy

A computerized search, including databases such as Web of Science, ScienceDirect, China National Knowledge Infrastructure (CNKI), PubMed, and Wanfang Database, focused on randomized controlled trials regarding curcumin supplements and human skeletal muscle injury in sports science. The literature search was updated until June 23, 2023, ensuring the inclusion of the latest research in our analysis. The authors assessed the PROSPERO database (https://www.crd.york.ac.uk/PROSPERO/) for published or ongoing research related to the title to prevent duplication. The result indicated no ongoing or published articles in this area.

### Data extraction

Two researchers extracted data using a double-blind approach. Information included the first author, publication year, sample size, gender and age of subjects, intervention details, and outcome indicators.

### Quality assessment

Quality was assessed using Cochrane guidelines, evaluating six indicators: random allocation method, allocation concealment, blinding, completeness of outcome data, selective reporting, and other sources of bias. Categorization was based on the number of indicators with low risk of bias.

### Statistical analysis

RevMan 5.3 software was used for statistical analysis, including relative risk and mean difference with 95% confidence intervals. Models were selected based on heterogeneity among studies, with sensitivity analysis and publication bias testing conducted.

## Results

### Literature screening process and results

**Fig 1:**
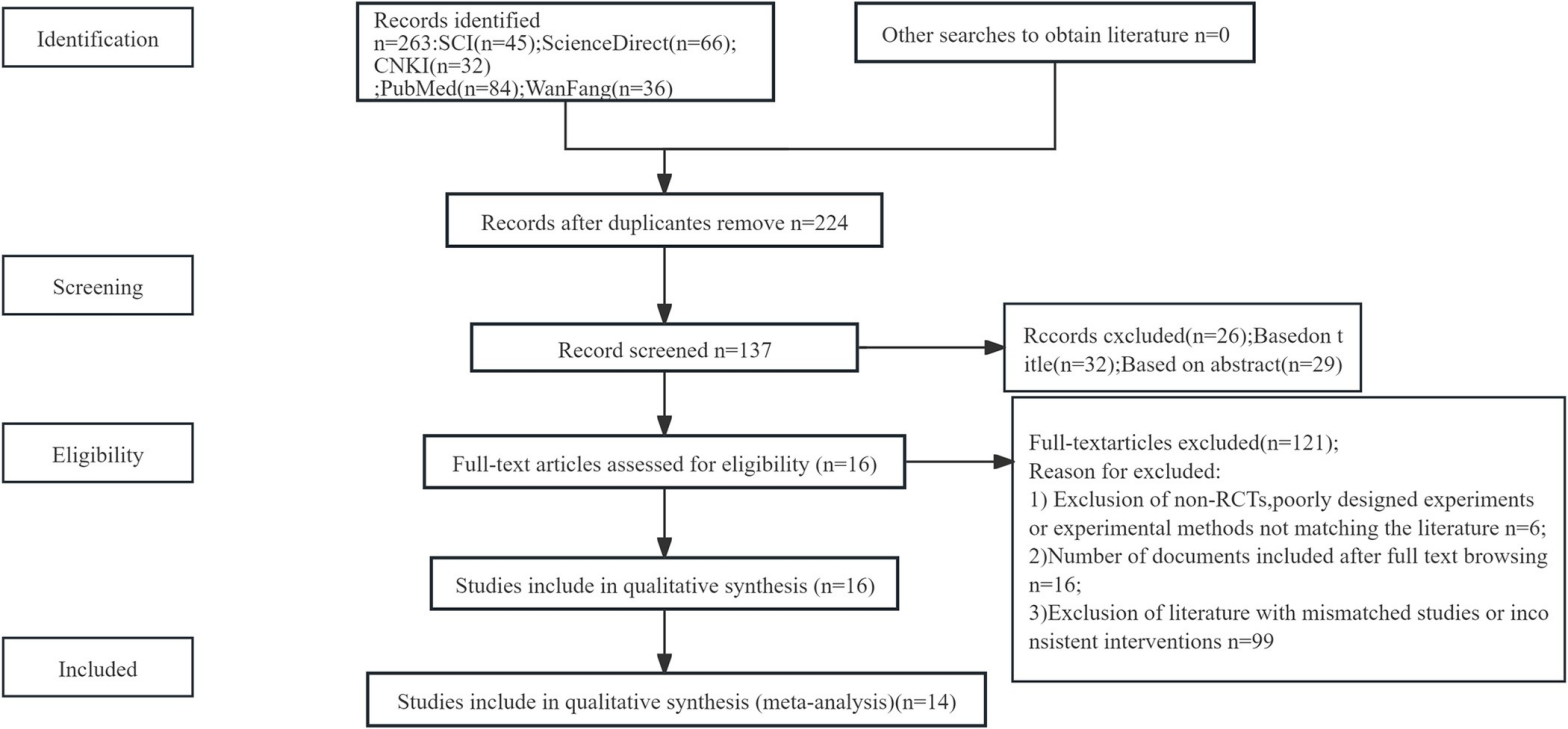
A schematic representation of the process incorporated in the literature.

Out of 263 articles, 14 were included in this meta-analysis. See (Fig 1) for details.

#### Basic characteristics of included studies

12 articles were selected, with specific details on sample size, supplement dosage, and outcome indicators organized in Table 1.

**Table 1:** Basic Information on the included literature.

Order number	Author	Year	Sample size	Supplemental dose	Outcome indicators
1	Ghojazadeh, M [12]	2022	18	4g/day	②④
2	N, K. J. [13]	2021	36	1g/ day	②
3	Hillman AR [14]	2021	22	1g/ day	③④
4	Mallard AR [15]	2020	28	500mg/ day	②③④
5	Amalraj A [16]	2020	30	500mg/ day	③④
6	Jäger, R [17]	2019	42	200mg/ day	③
7	TANABE, Y [18]	2019	16	180mg/ day	①③④
8	Tanabe, Y [19]	2019	20	180mg/ day	①②③④
9	Tanabe, Y [20]	2015	28	150mg/ day	①②③④
10	McFarlin BK [21]	2016	28	200,400,1000mg/ day	②③④
11	Nicol LM [22]	2015	17	5000mg/ day	③④
12	Basham SA [23]	2020	20	1.5g/ day	③④
13	Drobnic F [24]	2014	19	2g/ day	③④
14	Nakhostin-Roohi [25]	2016	10	150mg/ day	③④

①ROM ②IL-6 ③Muscle Soreness ④CK

#### Methodological assessment of included literature

**Fig 2:**
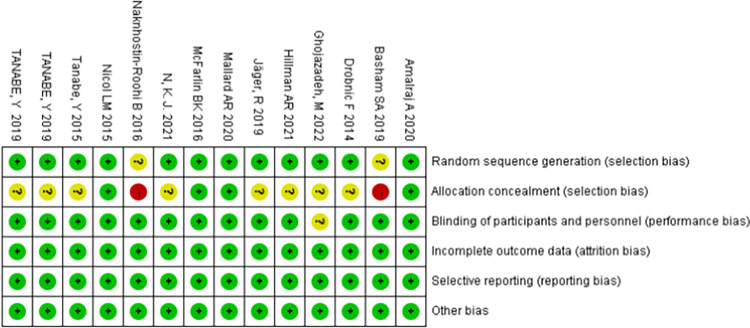
Schematic diagram of the methodological quality assessment in the literature. Category: "+" standard, "-" not standard, "?"NK.

**Fig 3:**
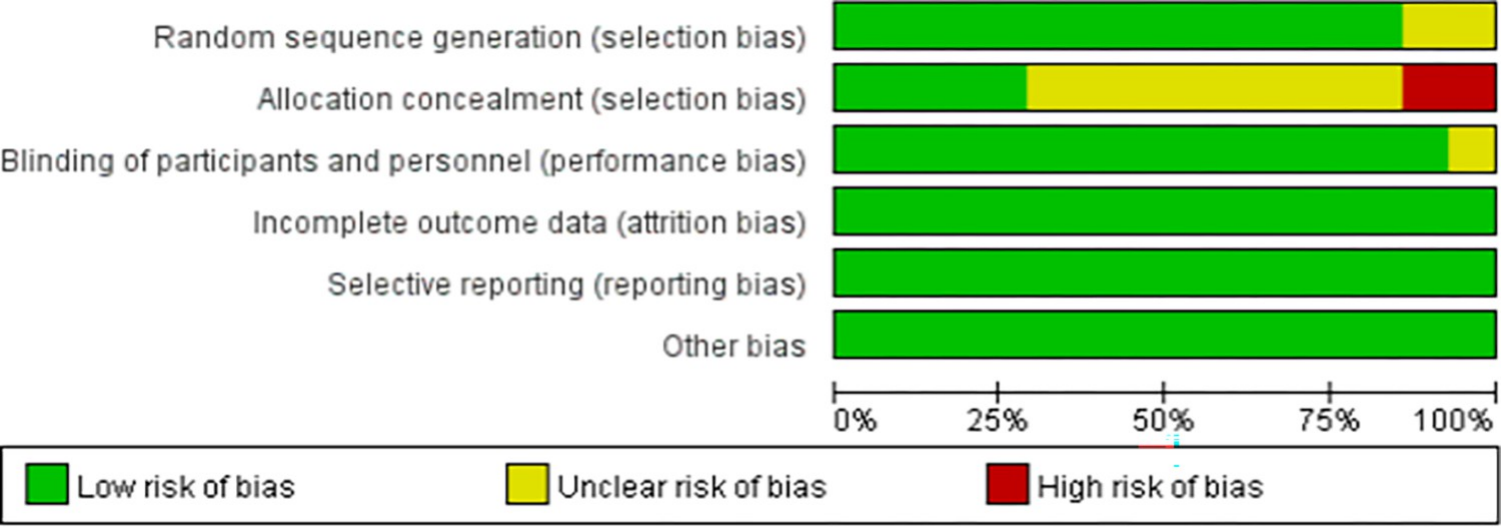
The portion of methodological quality assessment in the literature.

11 articles exhibited low risk of bias, while 3 were classified as medium risk. See Figs 2 and 3 for results of risk of bias assessment.

### Muscle soreness

#### Overall effect assessment

**Fig 4:**
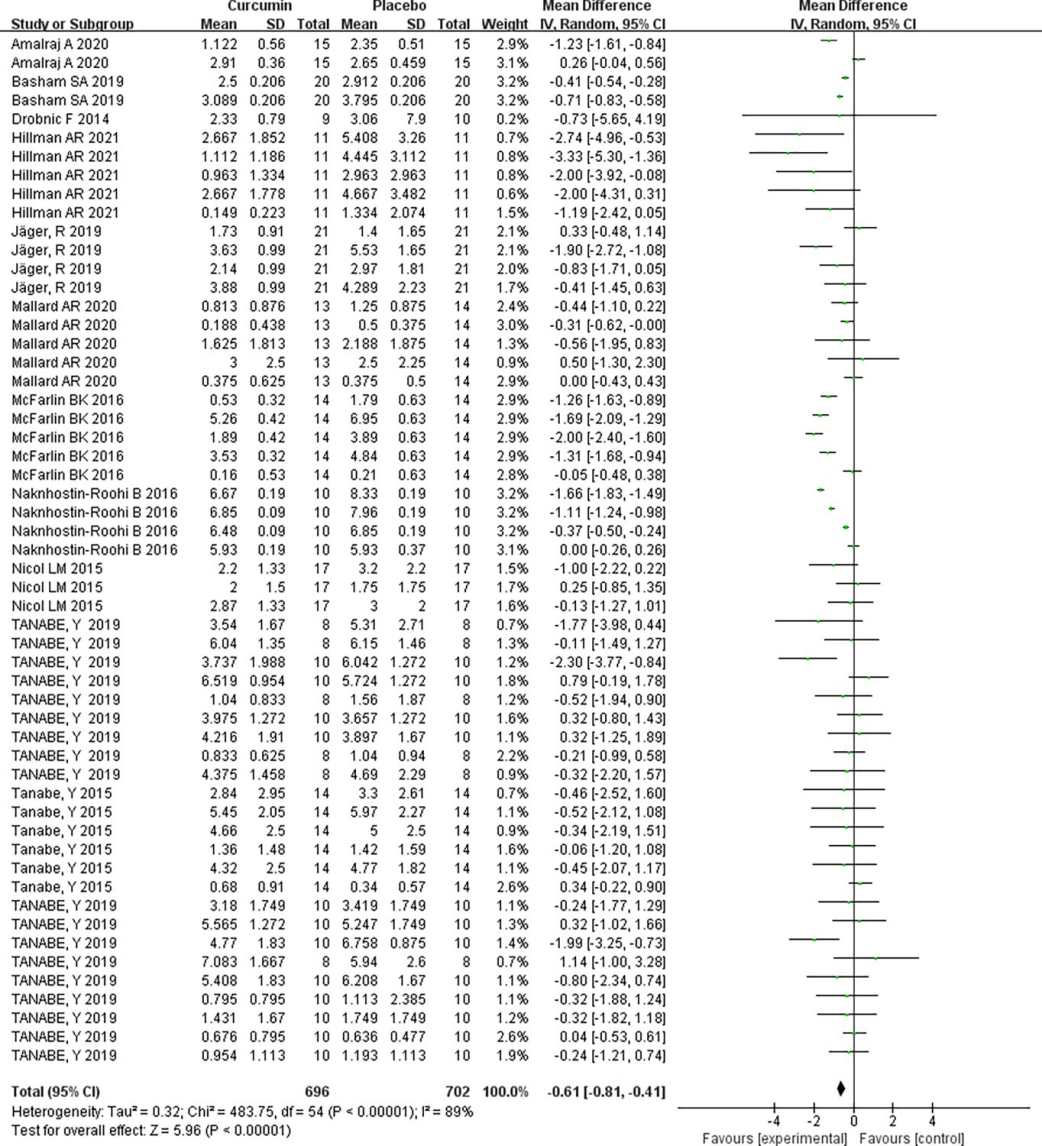
Forest plot of meta-analysis of curcumin supplementation for MMS relief.

An overall effect assessment encompassing the entire sample drawn from the 12 included papers reveals that curcumin supplementation effectively alleviates muscle soreness. The assessment of homogeneity (I^2^ = 89%, P < 0.00001) suggests significant variability among the studies, prompting the utilization of a random-effects model. The combined effect size for curcumin supplementation in mitigating muscle soreness registers at mean difference (MD) = -0.61, signifying a substantial reduction in pain, particularly in cases of lower back pain. The outcome of the two-tailed test (P < 0.00001) confirms the statistical significance of the combined statistic derived from multiple data sets. The corresponding 95% confidence interval, (-0.81, -0.41), underscores the effectiveness of curcumin supplementation in ameliorating muscular aches and pains, indicating a favorable outcome Table 2 and Fig 4.

**Table 2:** List of meta-analysis of curcumin on MMS relief supplemented in this study.

	Independent sample	Homogeneity test			Two-tailed test		Effect size and 95% confidence interval
		x^2^	P	I^2^	Z	P	
**Random effect model**	12	483.75	P<0.00001	89%	5.96	P<0.00001	-0.61[-0.81, -0.41]

#### Bias assessment

**Fig 5:**
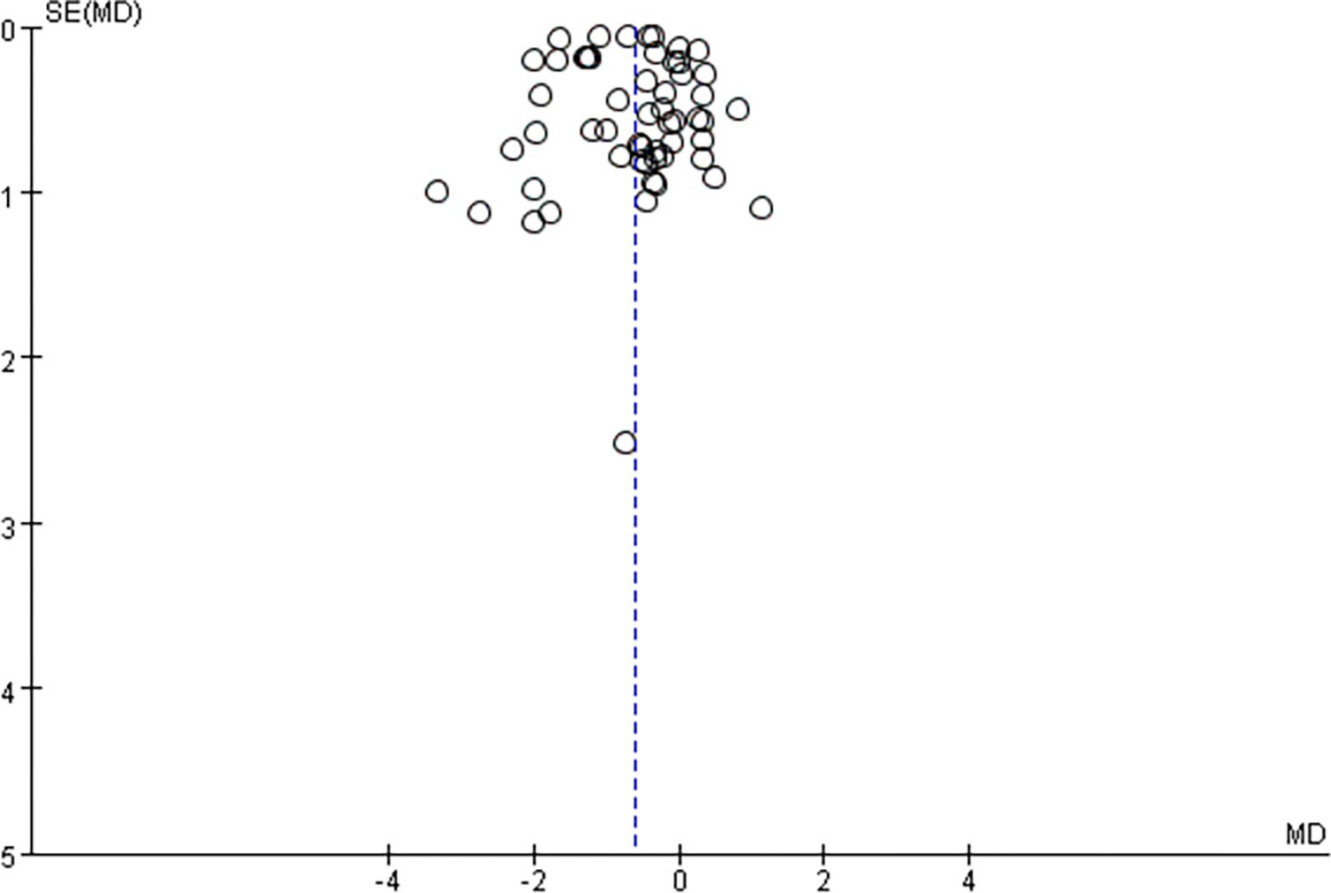
Funnel plot of Meta-analysis of the effect of curcumin on muscle soreness relief.

This Meta-analysis includes over 10 pieces of literature, making it suitable for bias assessment. As shown in Fig 5, the data points are mostly centered, with an even spread between the left and right. However, a few publications on the left exhibit slight bias. It should be noted that this bias is minor and is unlikely to significantly affect the results. This indicates the bias assessment is acceptable, with no apparent publication bias in the studies.

#### Moderating effects test

After the comprehensive overall effect test, this study acknowledges the potential influence of moderating variables. Thus, the investigation conducted hypothesis testing to examine the moderating factors that may affect the relationship between curcumin and the relief of muscle discomfort Table 3.

**Table 3:** List of regulatory effects tests in the meta-analysis of curcumin supplementation on muscle soreness relief in this study.

Moderator variable	Homogeneous variable			Form	Effect sizes and 95% confidence intervals	Two-tailed test		The number of literature	Sample size
	x^2^	P	I^2^			Z	P		
**Lead time**	30.52	P<0.0001	83.6%	pre-exercise	-0.12 [-0.46, 0.22]	0.69	0.49	9	251
				post-exercise	-0.38 [-0.83, 0.08]	1.63	0.10	7	209
				24H post-exercise	-0.67 [-1.19, -0.16]	2.56	0.01	9	257
				48H post-exercise	-0.81 [-1.36, -0.25]	2.83	0.005	10	276
				72H post-exercise	-0.81 [-1.27, -0.36]	3.51	0.0004	9	263
				96H post-exercise	-1.24 [-1.50, -0.99]	9.58	P<0.00001	5	142
**Whether trained**	0.07	P<0.00001	0%	trained	-0.57 [-0.82, -0.32]	4.46	P<0.00001	7	674
				untrained	-0.62 [-0.91, -0.32]	4.10	P<0.0001	5	704
**Intervention cycle**	2.71	P<0.00001	26.2%	single dose	-0.42 [-0.81, -0.02]	2.06	0.04	3	383
				one week	-0.56 [-0.93, -0.19]	2.99	0.003	6	657
				more than a week	-0.83 [-1.17, -0.50]	4.91	P<0.00001	3	358
**Supplemental dose**	1.86	P<0.00001	0%	<0.5g	-0.71 [-0.97, -0.44]	5.22	P<0.00001	7	1002
				0.5~1.5g	-0.53 [-0.84, -0.22]	3.32	0.0009	4	355
				>1.5g	-0.26 [-0.91, 0.40]	0.76	0.45	2	121

Subgroup analysis, based on time periods, reveals significant heterogeneity in effect sizes (I^2^ = 83.6%). This variability suggests that distinct time intervals exert unique influences on the relationship between curcumin and muscle soreness relief. The 96-hour post-exercise subgroup shows the most significant effect size (MD = -1.24, P<0.00001), followed by the 72-hour (MD = -0.81, p = 0.0004), 48-hour (MD = -0.81, p = 0.005), and 24-hour (MD = -0.67, p = 0.01) post-exercise subgroups. However, the effects were not statistically significant in pre-exercise (MD = -0.12, p = 0.49) and immediate post-exercise (MD = -0.38, p = 0.10) subgroups.

Subgroup analysis, categorized by training status, reveals no heterogeneity in effect sizes between the two groups (I^2^ = 0%). This observation indicates that the relationship between curcumin and muscle soreness remains consistently unaffected by individuals’ training status.

In the subgroup analysis considering intervention periods, mild heterogeneity is observed among the three groups (I^2^ = 26.2%) regarding muscle soreness relief. The group with an intervention period exceeding one week exhibits the most significant effect size (MD = -0.83, P<0.00001), followed by the group with an intervention period of no more than one week (MD = -0.56, P = 0.003). The group receiving a single dose demonstrates the smallest effect size (MD = -0.42, P = 0.04).

Subgroup analysis based on supplemental dosage indicates no heterogeneity among the three groups concerning muscle soreness relief (I^2^ = 0%). This finding implies that the supplement’s dose does not significantly impact the relationship between curcumin and muscle soreness.

### CK levels

#### Overall effect assessment

**Fig 6:**
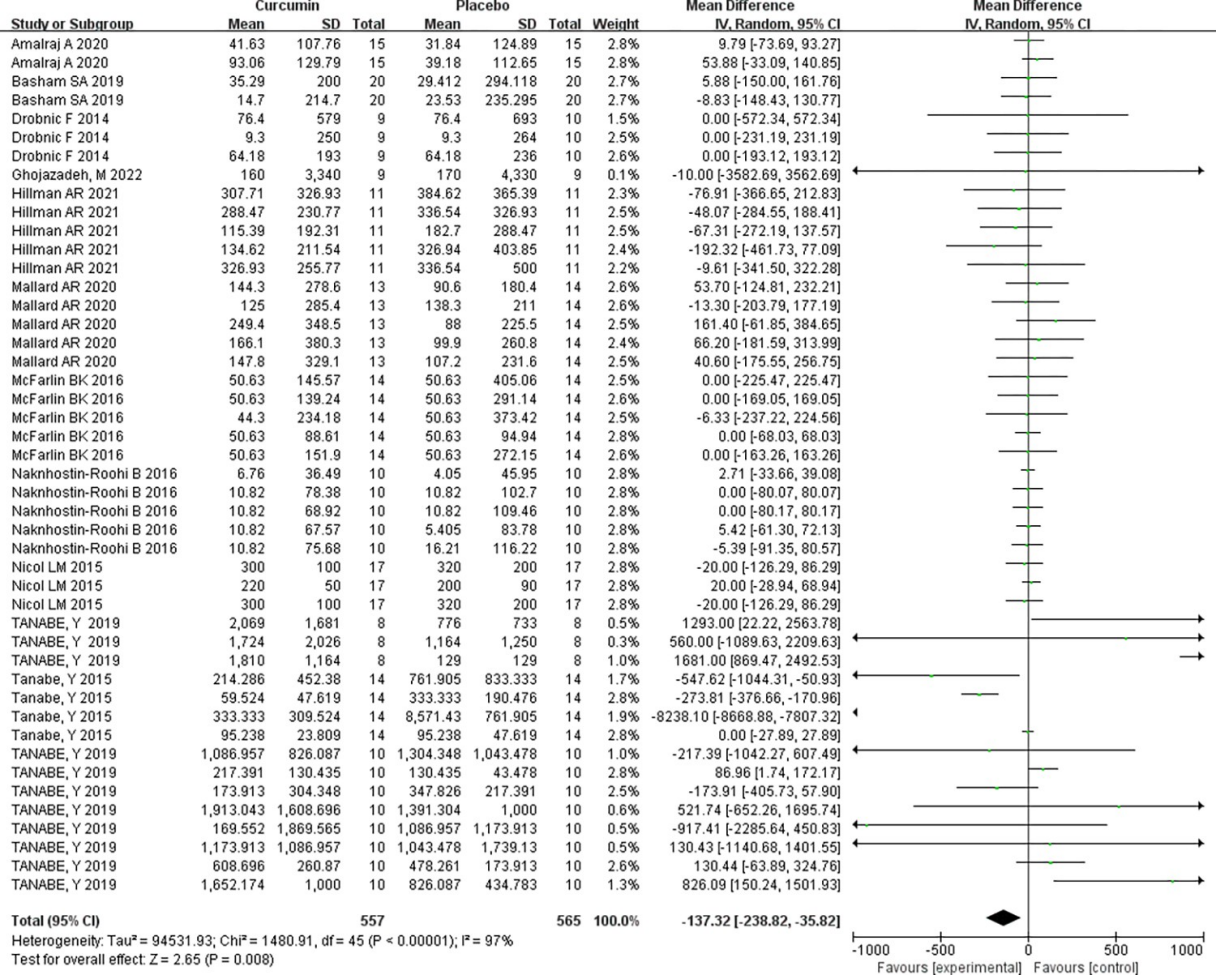
Supplementary meta-analysis of the effect of curcumin on reducing in vivo CK levels in forest fig.

A comprehensive analysis of the entire sample from 12 papers underscores curcumin supplementation’s effectiveness in significantly reducing in vivo CK levels. The homogeneity assessment (I^2^ = 97%, P<0.00001) reveals substantial heterogeneity among multiple studies, requiring a random-effects model application. The combined effect size for curcumin supplementation in reducing in vivo CK levels stands at MD = -137.32, signifying a meaningful reduction in CK levels. The two-tailed test results (P<0.008) validate the statistical significance of the combined statistic derived from multiple datasets. The corresponding 95% confidence interval, (-238.82, -35.82), emphasizes curcumin efficacy in achieving this outcome Table 4 and Fig 6.

**Table 4:** This study was supplemented with curcumin supplementation to reduce CK levels in vivo.

	Independent sample	Homogeneity test			Two-tailed test		Effect size and 95% confidence interval
		x^2^	P	I^2^	Z	P	
**Random effect model**	12	1480.91	P<0.00001	97%	2.65	0.008	-137.32 [-238.82, -35.82]

#### Bias assessment

**Fig 7:**
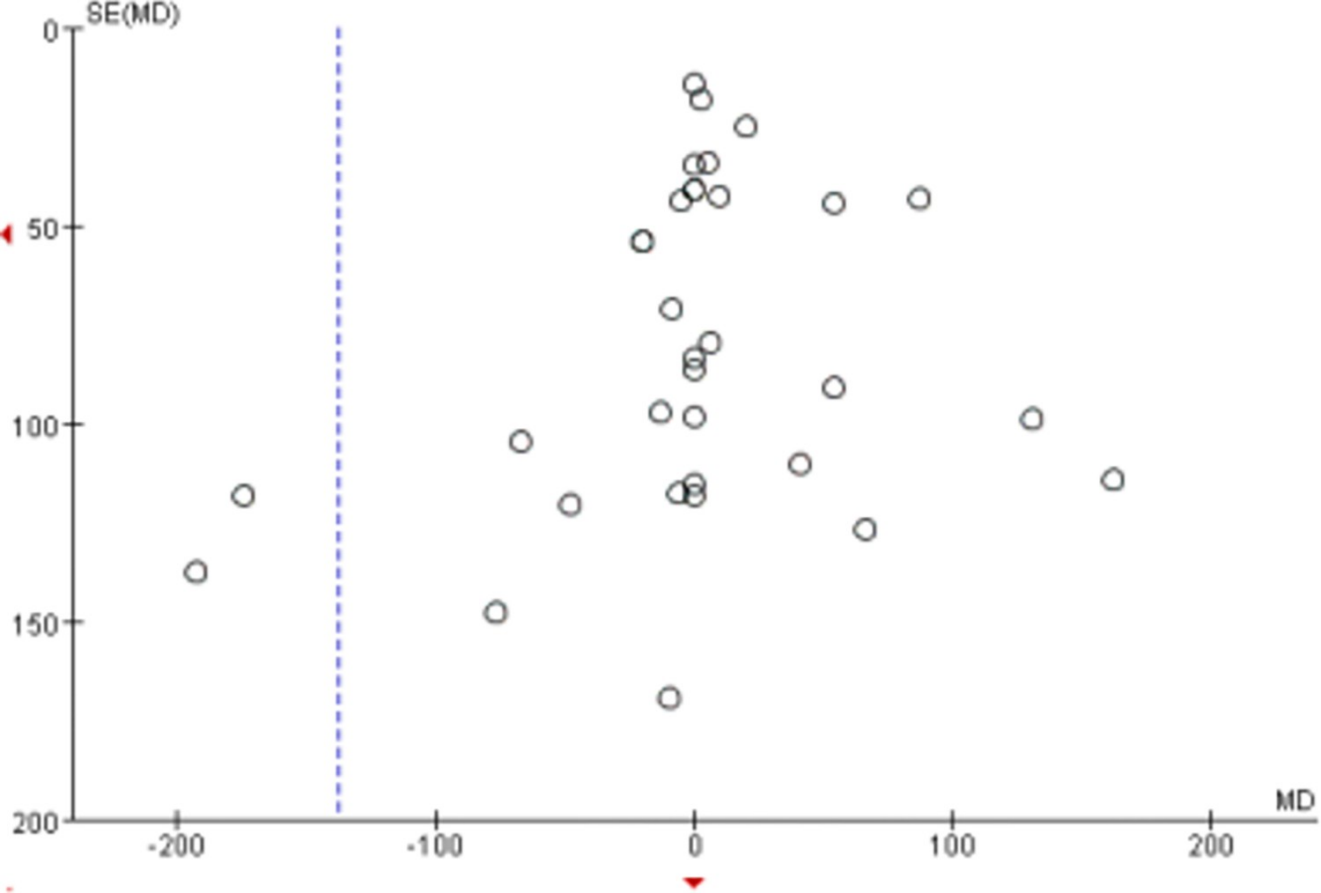
Meta-analysis of the effect of curcumin on reducing CK levels in vivo.

Including over 10 primary sources in this meta-analysis enabled bias analysis. Fig 7 suggests a slight bias tendency towards the upper end while achieving a balanced representation on the left and right. Some sources exhibit biased tendencies on the left side, but the impact on the results is minimal and acceptable. Therefore, the analysis confirms no significant publication bias among the studies.

#### Moderating effects test

Having identified the likelihood of potential moderating variables based on the overall effect test, this study proceeded to perform hypothesis testing to assess the influence of these moderating variables on the relationship between curcumin and muscle soreness Table 5.

**Table 5:** List of regulatory effect tests in the meta-analysis of the effect of curcumin supplementation in reducing CK levels in this study.

Moderator variable	Homogeneous variable			Form	Effect sizes and 95% confidence intervals	Two-tailed test		The number of literatures	Sample size
	x^2^	P	I^2^			Z	P		
**Lead time**	1.02	0.96	0%	pre-exercise	7.75 [-20.74, 36.24]	0.53	0.59	8	204
				post-exercise	-1.87 [-52.90, 49.16]	0.07	0.94	6	150
				24H post-exercise	8.32 [-15.51, 32.16]	0.68	0.49	9	218
				48H post-exercise	3.93 [-127.99, 135.84]	0.06	0.95	8	215
				72H post-exercise	-18.00 [-100.53, 64.54]	0.43	0.67	8	221
				96H post-exercise	-1709.11 [-6251.47, 2833.25]	0.74	0.46	4	114
**Whether trained**	2.40	0.11	59.9%	trained	11.22 [-17.59, 40.03]	0.76	0.45	7	562
				untrained	-282.55 [-455.29, -109.82]	3.21	0.001	5	560
**Intervention cycle**	22.37	P<0.0001	91.1%	single dose	-519.29 [-744.54, -294.03]	4.52	P<0.00001	3	347
				one week	22.73 [-19.38, 64.84]	1.06	0.29	7	585
				more than a week	-37.45 [-114.39, 39.48]	0.95	0.34	2	190
**Supplemental dose**	9.86	0.007	79.7%	<0.5g	-243.27 [-396.05, -90.49]	3.12	0.002	6	670
				0.5~1.5g	-0.53 [-0.84, -0.22]	3.32	0.0009	4	355
				>1.5g	7.48 [-31.95, 46.91]	0.37	0.71	3	177

No significant variation in effect size was observed among the six groups (I^2^ = 0%) in the analysis of measurement timing. This suggests that the timing of measurements does not impact the relationship between curcumin and CK levels.

Moderate heterogeneity was detected within the trained and untrained groups (I^2^ = 59.9%) in the analysis of the reduction of CK levels. This implies that an individual’s training status influences the relationship between curcumin and CK levels. The untrained group exhibited the most substantial effect size (MD = -282.55, P<0.001), while the trained group showed no significant effect (MD = 11.22, P = 0.45).

A high degree of heterogeneity (I^2^ = 91.1%) emerged among the three groups in the analysis of the reduction of CK levels based on the intervention cycle. This suggests that the intervention cycle significantly affects the relationship between curcumin and CK levels. Further analysis revealed that only the single-dose group showed a significant effect (MD = -519.29, P<0.00001), while the effect was not significant in the group with an intervention period of ≤1 week (MD = 22.73, P = 0.29) or the group with an intervention period of >1 week (MD = -37.45, P = 0.34).

The analysis of the reduction of CK levels based on supplemental dosage revealed a high degree of heterogeneity (I^2^ = 79.7%) among the three groups. This suggests that supplemental dosage impacts the relationship between curcumin and in vivo CK levels. The group receiving <0.5g exhibited the most substantial effect (MD = -243.27, P = 0.002), followed by the 0.5–1.5g group (MD = -0.53, P = 0.0009), while the effect was not significant in the >1.5g group (MD = 7.48, P = 0.71).

### ROM analysis

**Fig 8:**
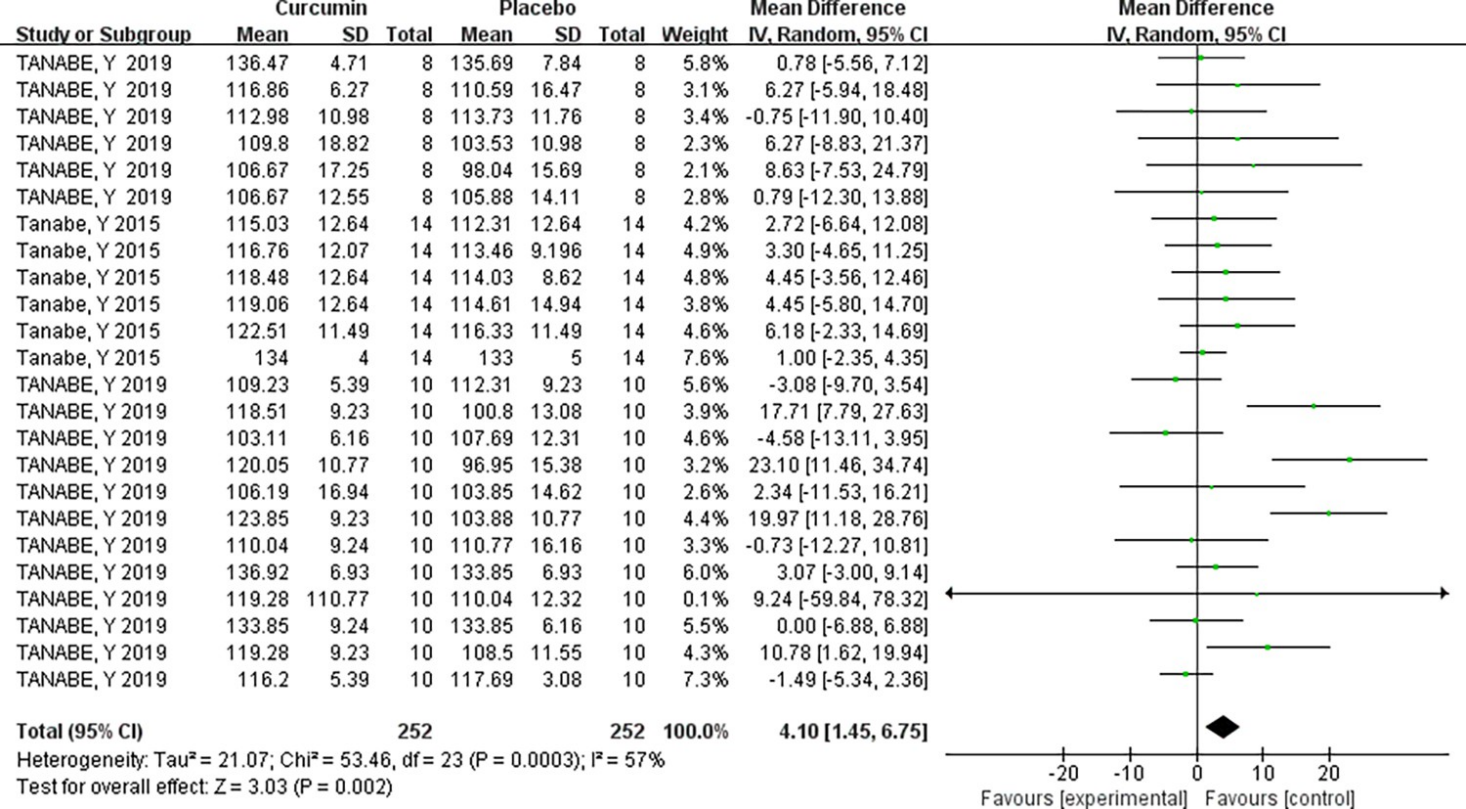
Supplementary meta-analysis of curcumin on enhanced ROM effect forest plot.

A comprehensive analysis of all samples from the three papers revealed that curcumin supplementation significantly enhances ROM. However, there was heterogeneity among multiple studies (I^2^ = 57%, P = 0.0003), necessitating the use of a random-effects model. The combined effect size for curcumin supplementation in enhancing ROM was MD = 4.10, indicating a significant improvement. The two-tailed test (P = 0.0002) confirmed the statistical significance of the combined statistic derived from multiple datasets, with a 95% confidence interval of (1.45, 6.75). These results strongly support the effectiveness of curcumin supplementation in enhancing ROM, see Table 6 and (Fig 8).

**Table 6:** Table of meta-analysis of the efficacy of curcumin on enhanced ROM.

	Independent sample	Homogeneity test			Two-tailed test		Effect size and 95% confidence interval
		x^2^	P	I^2^	Z	P	
**Random effect model**	3	53.46	P = 0.0003	57%	3.03	P = 0.0002	4.10[1.45,6.75]

### IL-6 analysis

**Fig 9:**
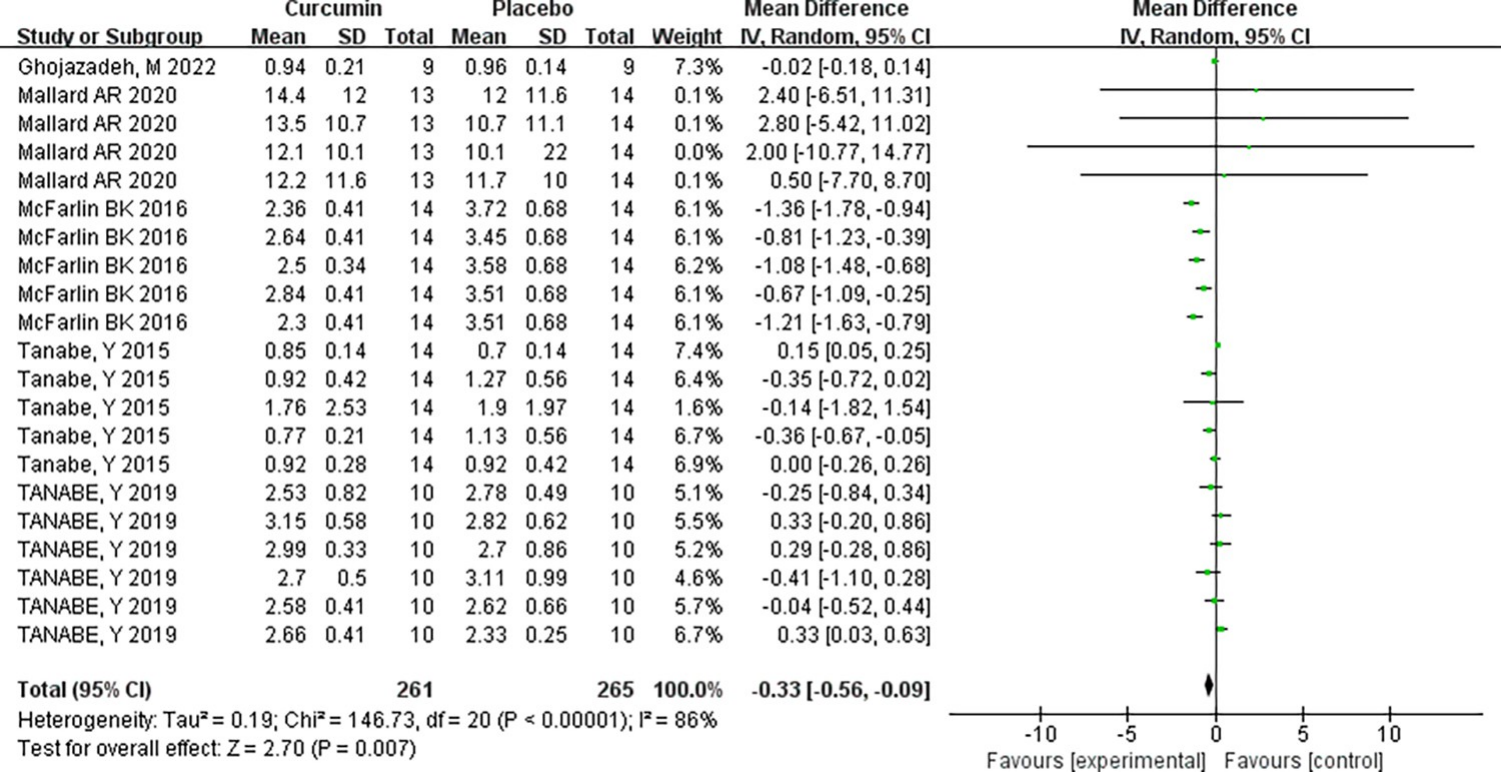
Supplement with a Meta-analysis of curcumin effect on reducing IL-6 forest plot.

A comprehensive analysis of all samples from the five papers revealed that curcumin supplementation reduced IL-6. However, there was heterogeneity among multiple studies (I^2^ = 86%, P<0.00001), necessitating the use of a random-effects model. The combined effect size for curcumin supplementation in reducing IL-6 levels was MD = -0.33, indicating a substantial improvement. The two-tailed test (P = 0.007) confirmed the statistical significance of the combined statistic derived from multiple datasets, with a 95% confidence interval (-0.56, -0.09). These findings strongly support the efficacy of curcumin supplementation in reducing IL-6, see Table 7 and (Fig 9).

**Table 7:** This study supplements a summary of the meta-analysis of the effect of curcumin on reducing IL-6.

	Independent sample	Homogeneity test			Two-tailed test		Effect size and 95% confidence interval
		x^2^	P	I^2^	Z	P	
**Random effect model**	5	146.73	P<0.00001	86%	2.70	P = 0.007	-0.33[-0.56,-0.09]

## Discussion

Curcumin’s potential impact on chronic inflammatory conditions like metabolic syndrome, arthritis, and cancer has been thoroughly researched in recent decades. Simultaneously, interest has grown in uncovering its role in EIMD. Notably, Vane et al. [26] identified curcumin’s NSAID-like effects, reducing pain and inflammation by down-regulating cytokines and prostaglandins, suggesting its promise in alleviating EIMD and Delayed Onset Muscle Soreness (DOMS). In this study, we considered changes in muscle soreness severity, ROM, and inflammatory markers as indicators of EIMD extent [27]. These parameters were used to assess curcumin’s potential benefits.

CK serves as a reliable marker of EIMD, and our analyses consistently demonstrated curcumin supplementation’s significant impact on reducing CK levels in the bloodstream. This effect is attributed to various mechanisms by which curcumin operates. Firstly, curcumin acts as a membrane protector by inducing structural modifications in cellular membranes [28], thus enhancing membrane integrity and reducing CK leakage into the bloodstream [29]. Additionally, research by MORIYUKI K et al [30] revealed that curcumin mitigates CK activity by reducing histamine and prostaglandins production and inhibiting COX-2, ultimately decreasing vascular permeability and reducing CK flow. Moreover, TAKAHASHI M et al [31] found that curcumin’s potent antioxidant properties enable it to neutralize ROS generated within the oxidative phosphorylation chain. These combined mechanisms contribute to curcumin’s effectiveness in reducing CK levels. Subgroup analyses showed curcumin supplementation was more effective at reducing CK levels in untrained individuals. This discrepancy might result from initially elevated CK levels in trained individuals, leading to relatively less pronounced reductions.

Curcumin alleviates muscle soreness through multiple mechanisms, notably by inhibiting COX-2 expression, which reduces CK activity and prostaglandin release, thereby easing soreness [32]. Meta-analyses have shown curcumin’s efficacy in reducing post-exercise muscle soreness. Studies [18,19] emphasize that the timing is critical, Whether taken before or after exercise, curcumin consistently reduces muscle soreness. For instance, individuals who consumed 150 mg of curcumin immediately after squatting experienced lower Visual Analogue Scale (VAS) scores for soreness at 48 and 72 hours compared to the placebo. Furthermore, supplementation after 4 days post-exercise significantly reduced soreness. Nakhostin, B et al [25] reinforced these findings, showing that 7 consecutive days of curcumin supplementation led to significantly lower muscle soreness in untrained individuals 3–6 days post-eccentric exercise. However, not all studies demonstrated significant reductions. Jäger, R et al [17] found that athletes taking 200 mg/day of curcumin for 56 days experienced soreness reductions compared to the placebo and low-dose (50 mg) groups, but these reductions were not statistically significance. Similarly, Tanabe, Y et al [20] found no significant improvements from curcumin consumption before and 12 hours after eccentric exercise. These varying results may be influenced by timing, dosage, and individual variability. Our analysis underscores curcumin’s ability to reduce CK levels and its potential to alleviate muscle soreness. Furthermore, our subgroup analyses highlighted that curcumin was most effective 96 hours post-exercise, suggesting a synergistic relationship between the body’s intrinsic recovery mechanisms and curcumin’s effects. Additionally, it implies that curcumin may have a prolonged presence in the body, contributing to its sustained impact. Supplementation for more than a week yields the most significant benefits. It’s essential to note that the validity of measuring muscle soreness can vary, as evident from the divergence between the VAS and Subjective Pressure Pain Threshold Test (PPT) [33]. This divergence emphasizes the importance of employing multiple assessment methods to enhance result accuracy.

IL-6, a versatile cellular messenger, plays a key role in various processes like lymphocyte activation, cortisol secretion, and acute-phase protein induction. Our meta-analysis indicated curcumin supplementation subtly reduces IL-6 levels. However, Barros et al [34] found that IL-6 concentrations increased immediately after a half-marathon, seemingly unaffected by curcumin supplementation. Likewise, TAKAHASHI M [35] reported no significant changes in IL-6 concentrations following a 2-hour cycling session with emergency curcumin supplementation. These findings have sparked scientific controversy. Cho et al [36] presented evidence that curcumin’s anti-inflammatory effects result from NF-kB down-regulation, inhibiting IL-6 and TNF-α expression. Additionally, a rat study [29] demonstrated curcumin’s ability to reduce TNF-α and IL-6 concentrations post-eccentric exercise, aiding muscle recovery. This conflicting data prompts further exploration of potential variables at play.

Curcumin’s efficacy in reducing inflammation might depend on the intensity of the inflammatory process. Curcumin seems most effective in reducing IL-6 chronic inflammatory diseases [36]. Additionally, IL-6 release post-exercise may be more influenced by carbohydrate intake rather than muscle damage [37]. This complex interactions between curcumin, IL-6, and other physiological factors requires a nuanced understanding. Further research is needed to unravel these interactions comprehensively.

Our meta-analysis highlighted curcumin’s impact on improving ROM. In a series of studies by Tanabe, Y et al [18–20], curcumin’s positive effect on ROM was evident. One study showed substantial ROM improvement three to four days post-exercise after ingestion 180 mg of curcumin within four days of exercise. Another study showed significant ROM improvement after 180 mg of curcumin post-exercise. In a separate study, notable ROM enhancements occurred at 24, 48, 72, and 96 hours after supplementing with 50 mg of curcumin before and after 150 centrifugal contractions of the elbow flexors. This effect attributed to curcumin’s ability to impede the NF-κB pathway, which mitigates inflammation and swelling, improving joint mobility and reducing stiffness [38]. NF-κB regulates myokines and plays a key role in orchestrating post-exercise inflammatory responses [39]. Excessive exercise can cause soft tissues stress, leading to bone and cartilage matrix fragments generation. These fragments activate NF-κB, resulting in cytokine secretion like IL-6 and TNF-α, causing tissue damage [40]. This elucidates the multifaceted dynamics at play, with curcumin’s capacity to quell the NF-κB pathway standing as a pivotal mechanism underpinning its ability to enhance ROM. The alleviation of inflammatory responses and the reduction of swelling not only contribute to improved joint mobility but also help mitigate stiffness, thereby enhancing overall ROM. This discovery underscores the potential of curcumin supplementation as a valuable adjunct in promoting post-exercise recovery, particularly concerning joint flexibility and mobility. However, further investigations are warranted to delve deeper into the intricate molecular pathways and explore potential variations based on individual factors.

Curcumin’s antioxidant efficacy depends on variables like exercise intensity and quantity in humans [41,42]. This analysis explored curcumin’s effects within a dose range of 150 mg to 4 g/day. However, the meta-analysis is not without limitations. First, it encompasses a limited selection of literature, potentially excluding relevant studies and introducing heterogeneity. Second, inflammatory marker responses and their relationship to exercise are contentious [43]. Further research is needed to understand the impact of inflammatory marker responses on post-training inflammation and curcumin supplementation on interleukins and other markers. Finally, due to available data constraints, we examined only four moderating variables. Future research should investigate a broader array of factors to deepen our understanding.

## Conclusions

This meta-analysis has shown that curcumin supplementation can significantly reduce skeletal muscle damage. Specific improvements were observed in markers such as CK levels, muscle soreness, IL-6 levels, and ROM. The findings suggest that the dosage and timing of curcumin supplementation are critical factors influencing its efficacy. Optimal results were seen with prolonged supplementation, especially in untrained individuals or those with less frequent exposure to muscle-damaging exercise. Given curcumin’s promising results in the context of exercise-induced muscle damage, further research is warranted to explore its benefits in other areas of health and disease, particularly in chronic inflammation and pain management.

For different curcumin effects, the optimal supplement plan varies. To improve muscle soreness and ROM, it is recommended to start a daily low-dose supplement (< 0.5g) one week in advance. To reduce CK and IL-6 levels, a low-dose supplement immediately after exercise can achieve the best results. Overall, we hypothesize that in any case, low-dose curcumin supplementation is most effective in alleviating EIMD. When the dose reaches 0.5g or higher, curcumin may act more on other signaling pathways, reducing its effectiveness in alleviating EIMD.

## Supporting information

S1 File Detailed description of the literature included. (XLSX)

S2 File The CK values included in the articles. (XLSX)

S3 File The IL-6 values included in the articles. (XLSX)

S4 File The ROM values included in the articles. (XLSX)

S5 File The muscle soreness values included in the articles. (XLSX)

S6 File The PRISMA 2020 checklist. (PDF)
